# The burden of narcolepsy symptoms from patients’ perspective: a narrative qualitative study

**DOI:** 10.1007/s11325-025-03407-y

**Published:** 2025-07-02

**Authors:** Jan Hlodak, Andrea Madarasova Geckova, Zuzana Dankulincova Veselska, Eva Feketeova

**Affiliations:** 1 Faculty of Social and Economic Sciences, Institute of Applied Psychology, Comenius University, Bratislava, Slovakia; 2 Faculty of Medicine, Department of Health Psychology and Research Methodology, Pavol Jozef Safarik University in Kosice, Kosice, Slovakia; 3 Faculty of Medicine, Department of Neurology, Pavol Jozef Safarik University in Kosice, Kosice, Slovakia; 4 University Hospital of L. Pasteur, Kosice, Slovakia

**Keywords:** Narcolepsy, Excessive daytime sleepiness, Cataplexy, Patients’ perspective, Qualitative research, Narrative Semi-structured interviews

## Abstract

**Background:**

Narcolepsy is characterized by excessive daytime sleepiness (EDS) and, in type one, cataplexy, often accompanied by disrupted nocturnal sleep, parasomnias, and other impairments. This study aims to explore patients’ perceptions and emotional experiences of these symptoms.

**Methods:**

A qualitative study was conducted using narrative semi-structured interviews with 25 narcolepsy patients, guided by a structure approved by a multidisciplinary expert board. The interviews, conducted at the patients’ homes or hospitals, were transcribed, inductively analyzed to develop a codebook, and subjected to thematic analysis.

**Results:**

The onset and manifestation were key themes for each symptom. EDS was often described as “microsleeps” or “shutting off,” causing embarrassment when occurring publicly and influenced by factors such as the weather, seasons, or menstruation. Cataplexy was commonly triggered by positive emotions or situations, including sexual intercourse, with patients often suppressing emotions to prevent episodes, leading to misinterpretations by others as drunkenness. Nocturnal sleep disturbances, including vivid dreams, nightmares, sleep paralysis, and hallucinations, led to negative emotional experiences. Overall, narcolepsy symptoms significantly impacted patients’ emotional well-being.

**Conclusion:**

The burden of narcolepsy is deeply tied to patients’ perceptions and emotional experiences, significantly affecting their quality of life, yet few studies have used qualitative approaches to explore their perspectives.

## Introduction

Narcolepsy is a chronic neurological disease. According to the third edition of the International Classification of Sleep Disorders (ICSD-3-TR), it is categorized as a central disorder with hypersomnolence [1, 2] and manifests by excessive daytime sleepiness (EDS) with the inability to stay awake and a subjective feeling of sleepiness [3]). The episodes of EDS usually happen in times of inactivity or during a monotonous situation, but also during active situations, e.g., during a conversation or when writing [4]. This symptom occurs in both narcolepsy type one (NT1) and type two (NT2). NT1 is manifested by attacks of cataplexy in comparison with NT2, which is without cataplexy. Cataplexy can be described as an episodic loss of muscle tone with persisting wakefulness; it is usually triggered by a strong experience of usually positive emotions and the consciousness stays preserved [3]. It is typically partial, affecting a certain body part (e.g., the face, head, neck, hand or legs, etc.). Rarely, it manifests as a complete collapse of the body, during which every skeletal muscle loses tone except the diaphragm and ocular muscles [3, 4].

According to Barker et al. [5], patients with narcolepsy also suffer from concentration and cognitive difficulties as well as daytime fatigue. Clinical features of the disease also show other manifestations, such as sleep paralysis (inability to move during a transition between sleep and wakefulness), hypnagogic and hypnopompic hallucinations (usually auditory, visual or tactile, happening before falling asleep or after waking up), disrupted nighttime sleep (fragmental and ineffective), automatic behavior (common mostly in children) and other symptoms, e.g., vivid or lucid dreaming, nightmares, etc. Only EDS is always present in both types of narcolepsy [4].

The prevalence and incidence of the disease may vary based on location, but it is considered a rare disease, with about 30 people for 100,000 being diagnosed with narcolepsy [6]. According to Feketeova et al., [7], the prevalence of narcolepsy in the Slovak Republic ranges around 10.47 cases per million inhabitants, and the total number of patients diagnosed or treated at the time was 61. The disease itself is considered underdiagnosed or frequently misdiagnosed for the nature of the symptoms and their assessment. According to Kallweit et al. [8] suggest the number of questionnaires and screenings (e.g., Epworth Sleepiness Scale to measure EDS [9] or Swiss Narcolepsy Scale [10] to measure narcolepsy symptoms etc.) might not be as efficient in the symptom understanding and further validation is required. Lammers et al. [11] also suggest the necessity for diagnostic manuals revision. They also offer the diagnostics criteria, tests and terminology updates according to the new knowledge and their expertise.

Other important aspect of the diagnostics and treatment lays in the patients’ perspective. According to Bassetti et al. [12] this perspective is necessary for the development of good clinical practice and patient-oriented guidelines. They describe the recommendations of the patients including the use of plain language and understanding that the terminology used by the patient might differ from the one medical professionals’ use. This may lead the doctors to incorrect clinical picture and result in inaccuracies. They also recommend that medical doctors should use open ended questions to prevent misleading of the patients e.g., general questions not directly focusing on the exact symptoms’ manifestation. Additionally, the doctors should consider questions focused on other problems aside from EDS and cataplexy.

The manifestation of these symptoms may have a negative effect on a patient’s quality of life. Multiple studies focused on describing the impact of narcolepsy symptoms on patients’ psychosocial life (e.g., Wasling et al., [13]; Chin et al., [14]; Ong et al., [15] etc.) have mostly employed a quantitative study design. Only a few recent studies focused on the patients’ perspectives of the disease have used a qualitative research design (e.g., Schokman, et al., [16]; Franceschini et al., [17]; Tadrous et al., [18]); however, to the best of our knowledge, none of those qualitative studies focused on the complex subjective experience with symptoms of narcolepsy from the perspective of patients.

Therefore, this qualitative study aims to better understand and describe how patients with narcolepsy perceive and emotionally experience the symptomatology based on interviews with a sample of Slovak narcolepsy patients with maximum heterogeneity. Understanding the perception of symptomatology may enhance the characterization of the narcolepsy clinical phenotype by incorporating patients’ subjective experiences.

## Methods

### Research background

The methodological background of this study was in line with the qualitative research methodology created by the Health Experience Research Group (HERG) from Oxford University focusing on the individual patients’ experiences [19–21]. This study is part of a larger project aimed at narcolepsy patients’ perspectives focused on the disease and the burden of living with this condition using narrative semi-structured interviews. The final outcome of the study is the creation of a public webpage of patients’ experiences with narcolepsy.

### Preparation and recruitment

This study was approved by the Ethics Committee of Pavol Jozef Safarik University in Kosice, Faculty of Medicine in Kosice, Slovakia, and by the Ethics Committee of Louis Pasteur University Hospital in Kosice, Slovakia. The protocol was listed under the name “Quality of life in patients suffering from narcolepsy (2022)” and approved under number 29 N/2022. Data were collected in compliance with the ethical principles outlined in the 1964 Declaration of Helsinki, including its subsequent amendments or equivalent ethical guidelines. Questions and topics for the narrative semi-structural interview were created based on a literature review and were supplemented and approved by a multi-disciplinary board composed of a psychiatrist, psychologists, qualitative researchers, a neurologist and somnologist, and narcolepsy patients.

During the first meeting, the board critically reviewed the proposed topics and questions, provided suggestions for additions, modifications, or removals, and were invited to submit further written revisions via email. After five interviews were completed, a second meeting was held to present the preliminary analysis, validate emerging themes, identify additional topics, and gather further expert input to refine the research framework.

This was implemented to ensure that every possible aspect of experiences related to narcolepsy, including relevant quality of life (QoL) domains, would be adequately addressed. After the preparation of the narrative semi-structured interview guide and ethics committee approval, the authors of this paper (EF, JH) started the recruitment process using purposive sample selection.

### Sample selection and characteristics

The research sample was recruited from the database of patients at the Sleep Laboratory of the Neurology Clinic of L. Pasteur University Hospital in Kosice. Every participant was addressed individually, either by phone, email, or onsite at the clinic. The sample selection for this research tried to comply with the condition of maximum sample variation (according to sex, age, education, region of living, living condition, marital status, employment status, etc.). Inclusion criteria were (1) the patient had to be 18 years of age or older, (2) had to understand and speak the Slovak language, and (3) must be diagnosed with NT1 or NT2 according to the criteria of ICSD 2nd or 3rd edition [1, 2], based on whether the patient was diagnosed before or after the release of ICSD-3. Informational pamphlets were handed over to all prospective respondents, and the purpose and outcomes of the research interview were explained. After the process of being informed, the volunteer patient signed an informed consent and willingly agreed to participate.

Therefore, 25 patients were interviewed and included in this study. Most of the sample were female (72%) and patients with NT1 (92%). The national prevalence caused the proportion of NT1 and NT2 patients in the sample. According to Feketeova et al. [7], 84% of Slovak patients suffer from NT1. 11 patients (44%) were medicated at the time of the interview. The majority of our interviewees were young or middle-aged adults, but we also interviewed two patients under 20 years of age and two elderly people. One of the patients was diagnosed a few days before the interview. Almost all the patients were Caucasians (96%). One was a member of a minority; therefore, the sample reflects the current socio-demographic population in Slovakia. The sample is described in detail in Table 1.

### Interview process

The interviews took about an hour, with the longest lasting more than an hour and a half. The process of interviewing started with an initial narrative question – *“Try to remember the time you or your close ones started to register something was wrong.”* – encouraging respondents to share with us their perspectives on narration of the disease. After the narrative monologue, a semi-structured interview continued. Interviews were administrated by the first author (JH, a psychologist trained in qualitative interviews) at the patients’ homes or at the sleep clinic. Each interview was audio recorded for transcription, but everyone was also recorded by a second audio or video recorder, depending on the patient’s preference. Later, a written consent for copyright was signed by the patient, asking them to allow a chosen format of record for later use, e.g., for the purpose of the webpage creation, for educational purposes, or for publications. The participants had the choice of selecting video, audio, or anonymous transcription of an interview to be used on a webpage and for research and other academic purposes. Each interview was transcribed verbatim, and every patient had a chance to choose segments of the text to not be used in later analysis or to change the name of a participant. Every participant permitted use of the transcripts in the secondary analyses, apart from the purpose of webpage use. Data collection took place from May 2023 to August 2024.

**Table 1:** Sociodemographic characteristics of the sample (*n* = 25)

Sociodemographic indicator	Specification of sample (*n* = 25)		Prevalence *n* (in%)
**Narcolepsy type**	NT1 [n female] NT2 [n female]		23 [17] (92 [68]) 2 [1] (8 [4])
**Sex**	Male		7 (28)
	Female		18 (72)
**Age**	Min–Max range Mean [SD]		19–77 40.04 [14.97]
**Date of Birth**	< 1950 1951–1960 1961–1970 1971–1980 1981–1990 1991–2000 > 2000		1 (4) 1 (4) 3 (12) 5 (20) 5 (20) 8 (32) 2 (8)
**Relationship status**	Single		9 (36)
	In a relationship		3 (12)
	Married		10 (40)
	Divorced		3 (12)
**Have children**	Yes		13 (52)
	No		12 (48)
**Employment status**	Student		2 (8)
	Employed		10 (40)
	Unemployed and w/o pension		2 (8)
	Disability pensioner or retired Pensioner or retired + working		9 (36) 1 (4)
	Maternity leave		1 (4)
Living location*	Village Town (< 45 000) City (> 45 000)		8 (32) 9 (36) 8 (32)

*According to the location status determined by the Municipal Organization Act or the number of residents

### Data analysis

Transcribed interviews were checked, and names and places were removed to ensure anonymity throughout the analysis. The first five interviews were coded inductively to find essential topics occurring in the interviews. These were later supplemented by additional codes after the team of analyzers consensually created a codebook. Each interview (the first five also) was later analyzed according to the codebook deductively using the MAXQDA software package for MAC (v.24) for easier coding procedure and segment classification. Therefore, an abductive method of narrative analysis was used. The abductive approach in analysis has multiple advantages, e.g., broadening existing understanding, deepening insights from the interviews, and providing more nuanced and accurate analysis as well as plausible explanations of specific phenomenon [22]. The narrative analyses approach also provided the opportunity for potential emerging narratives for the presentation of advanced results with new insights [23]. Every interview was coded independently by three authors of this paper (JH, AMG, ZD), and a consensual (collaborative) coding was later performed for each transcript again. Afterwards, specific coded segments were analyzed together based on the topic using thematic analysis [24] in the form of the OSOP (one sheet of paper) approach [19], in which all researchers were trained. This process involved systematically going through each section of selected segments and recording, on a single sheet of paper, all the different issues raised in the coded segments. The thematic map was created to describe all aspects of the topic mentioned in the interviews and to connect the identified subtopics. For this study, we used segments focused on each symptom of narcolepsy mentioned. Summaries of the findings also reflect the thematic maps and describe robust subtopics as well as rarely mentioned nuances.

## Results

Twenty-five text transcripts were used in the analysis for the purpose of this paper. We identified core thematic circles based on the symptomatology of narcolepsy together with several subthemes connected to each individual symptom, mostly focusing on the process of manifestation, influencing factors, changes of the manifestation through time, and emotional experiences behind the symptom. We have divided the themes according to the severity of manifestation in the sample.

### Excessive daytime sleepiness

#### Onset and manifestation

Excessive daytime sleepiness (EDS) can manifest suddenly, leaving patients unaware of its onset. Many patients described their episodes of EDS as “microsleeps” or refer to them as moments when they feel like they “shut off.” The sleepiness can often be severe and noticeable to those around them. One patient, a 54-year-old female with narcolepsy type one (*F54NT1*), recounted how others typically noticed her EDS when she began speaking off-topic during conversations. She described this as “just talking for the sake of talking”. Another patient also recalled a period when her EDS became so pronounced that she would fall asleep while eating, sometimes with a fork still in her mouth. On the other hand, some patients report being able to sense the onset of EDS. Many of them spoke of specific warning signs, such as unusual sensations in their eyes, incoherent speech, trembling, or tics, which they recognized as precursors to an EDS episode. Some patients also mentioned fatigue as a symptom of narcolepsy but usually confused the term with the manifestation of EDS. One patient explained the effect of EDS as something that can worsen fatigue even more after she wakes up from it, and it can be so pronounced that she cannot continue with work or do anything else but go to sleep.

“I’ve gotten used to it over the years; it’s just how it is for me. Yes, I do have it, but over the years, I’ve become accustomed to it. Many times, it overtakes me, and I fall asleep immediately, but other times, I can feel it coming. It’s like a fog, like a kind of hum, a gritty feeling in the eyes, as if someone were sprinkling ashes or sand on me. You feel it, and you’re trying to fight it, in such extreme fatigue. That’s like the first wave of fatigue, and I already sense it’s coming. I know now when it’s about to hit. It’s not constant, but it happens often, very frequently. So, I’ve adapted to it—when I start feeling it in my eyes, when my eyelids grow heavy, when the pressure starts to build, the headache begins, or those red dots appear, like when you stand up quickly from sitting. Or that tingling sensation in front of your eyes. When this happens, or that fog sets in—everyone might experience it differently—but for me, these are the signs. And I know, within 15–20 minutes, I’ll definitely collapse somewhere.” *Male*,* 39 years-old with narcolepsy type one (M39NT1)*.

In some patients, episodes of EDS are followed by memory lapses, where they cannot recall what they were doing or where they placed certain items. One patient often misplaces her phone, while another sometimes engages in phone conversations but later cannot remember who she spoke to or what they discussed. Many patients also report that EDS is uncontrollable, and they feel compelled to succumb to it. EDS can manifest in patients during various situations, e.g., monotonous, unengaging, or boring activities or situations. The most reported context for EDS was during travel on public transportation. For instance, some patients shared that they would fall asleep not only on the bus but also at the bus stop. One elderly patient experienced sleeping through an entire flight, including takeoff and landing. Many patients also noted that it was easiest to fall asleep while sitting.

“It happens mainly during monotonous activities, essentially anything where I am not actively engaged. Whenever I am sitting—whether it’s during a lecture, an exercise class, on a bus ride, or waiting in a doctor’s office—I fall asleep. During internships, when we had to sit in a clinic and listen, I would often fall asleep there as well. Anywhere I am sitting and not actively doing something, I fall asleep. Even while reading a book or watching TV—any kind of film or series—unless it is something very engaging, I eventually fall asleep after some time. Of course, there are times when I don’t fall asleep; I could read a book all day, but it must be something extremely captivating. Even then, if I start to feel sleepy, I must get up, walk around, or eat something to keep going. However, if I am just sitting still, it’s inevitable—I can’t help but fall asleep, no matter what I do.” (*F28NT1)*.

Overall, fatigue and EDS can impact basic activities of patients because of the instability of the energy provided to them during the day. These energy fluctuations can be judged as laziness by other people. One patient compared himself to an old battery that is easily drained, that is always working at only 30% and must recharge every hour for three to four hours.

#### Automatic behavior during EDS

During episodes of EDS, patients may perform various tasks, such as continuing to work on an assembly line or falling asleep while writing, only to continue typing while asleep. However, this activity is of poor quality and, in some cases, may even be dangerous and pose a threat to health. One patient described it as a state where “the body continues, but the brain is asleep.” Many patients who experienced EDS during their school years recalled engaging in automatic note-taking in class. However, these notes were nonsensical and often consisted of scribbles and illegible marks. One patient talked about the impact of this behavior at work.

“Because it’s impossible to work. I used to work in a factory that produced seals for household appliances, and everything was glued manually with adhesives. I would dip the rubber into glue and join it together. I could hold it for an hour, with my eyes closed, sleeping. If no one woke me up, I would either wake up on my own or keep going. I also had episodes where I could spend an hour working on a single rubber piece, but I would end up gluing it to my gloves and other places. Eventually, they moved me to work on a machine, and that became a problem because the machine required foot control to operate. It involved cutting rubber, so I could have easily placed my hands there and had them cut off. Sometimes I woke up in the middle of it, or someone would grab my shoulder because I was standing on the pedal. The machine would open and close repeatedly, and I wasn’t putting any rubber into it—nothing at all.” *(F49NT1a)*.

#### Factors influencing EDS

Patients described various influences (besides situations and activities) that have an impact on how often and how strongly EDS occurs. The effects of weather and seasons, higher stress or excitement, inhaling or exhaling air in a closed room were often described. For one female patient, EDS manifests itself when she relaxes her work performance and stops for a while. Drowsiness also affects her when she looks at the monitor or mobile phone for a long time.

“So, it’s kind of awkward because when it’s [manifesting] in front of people. That even if I have the pills, they don’t work 100%. For example, menstruation also affects it, great heat affects it when it’s like this in the summer, even when it’s raining. Unfortunately, the pills do not work 100%. Well, it’s the same with travelling.” *(F26NT1)*.

“In winter, it’s cold outside, it’s raining, I’m inside, so I don’t mind [sleeping]. But in the summer, I will still sleep. I would also like to go outside; I have a granddaughter, and I have someone to take care of, not to be with her again. […] I sleep more in winter. In winter, I also go to bed at five, half past five. In winter. But in the summer, in the summer I go to bed at nine, ten, eleven, but already in the morning, I must catch up, as I say. I no longer get up at six, but at nine, when I go to bed at eight in the evening, I get up at six, and at ten, I get up at eight. So, I need those nine to ten hours. But when I didn’t take medicine, I was able to sleep for fifteen hours. Fifteen hours at a time and during the day an hour, sometimes five. Limited to one to five hours, sometimes two hours, sometimes three hours.” *(F46NT2)*.

#### Changes of EDS in time

EDS does not always manifest the same for all patients and may change. For some, it is the first symptom of narcolepsy, and it may get worse. This can either be gradual, i.e., it lasts for a long time, or sudden and therefore immediately visible to the patient and his surroundings. One patient noticed a worsening of EDS when she was already sleeping 12 h a day and with 3 awake hours had to go to sleep again. In other patients who are being treated there is mostly an observable improvement in the manifestation of sleepiness. However, the treatment may not be effective for every patient. For some, there is no improvement or alleviating of the symptoms at all.

“You know what, I’m already retired [from work], so it’s better now; I have fewer narcoleptic attacks and cataplexy, because I’m at home [and] when I need to sleep, I start to yawn, but I can feel in my eyes that some kind of attack is coming. I go to bed, I sleep for an hour or two and I continue to function as if I gain strength, then I work for three or four hours and then I go to bed again for a bit, so take a nap, so it’s better now, but when I was working, it was hard.” *(F54NT1)*.

#### Emotional experience with EDS

The occurrence of EDS can have a significantly negative impact on patients with narcolepsy. This is the case because of its manifestation in public (e.g., at school or work), where others do not recognize this symptom and may confuse it with drunkenness. It also affects the social life of patients. This is why several respondents described being afraid of the prejudices that others may have about EDS. The symptom itself was described by most patients as unpleasant, especially because of the situations it manifests in. Many patients feel confused after falling asleep during the day; one of them said that at the time of EDS onset, he could not distinguish whether he was asleep or not. On the other hand, other patients said falling asleep because of EDS is pleasant because the tiredness leaves your body as a result.

“And for me, it is so uncomfortable to sit in the waiting room. In the meantime I fall asleep 10 times, then my head hurts. It has already happened to me that I did not wake up once, that I fell on another patient [next to her]. But when I come here [sleep clinic], everyone is nice to me, and everything is fine.” *(F26NT1b)*.

### Cataplexy

#### Onset and manifestation

Cataplexy is manifested by a sudden weakening of muscle tone. Patients stated the inability to maintain the stability of a certain part or even the whole body during such an attack. The muscles lose tension, and some patients stated they sometimes even fall to the ground. During this state, patients were almost always fully conscious but were only able to move their eyes and breathe while the body was generally weakened. However, in some patients, this condition is only partially manifested in certain parts of the body. One patient said that his partial cataplexy was manifested by drooping of the shoulders; another had very frequent head drops, which caused constant pain in her neck. On the other hand, some female patients could not control the muscles of their jaw during an attack, which manifests in poor articulation, one felt tics in her face and another mentioned that during cataplexy she made various grimaces and twisted her mouth.

“[…] mostly like cataplexy attacks, they don’t have to manifest themselves completely on the outside, as if you can also have them internally, you feel as if your internal weakness, and when you stop it for a while […] the emotions, so you don’t have to show it at all, or you change, you just change the topic completely, but of course, [I am] not only going to my knees but even lay down. So, fair sex can get you to such a stage without problems.” *(M40NT1)*.

Some patients are unable to predict when a cataplectic attack will occur, and it can happen suddenly and without warning, leaving them unprepared. However, some individuals reported being able to sense precursors to an attack, allowing them to take preventive measures, such as sitting or lying down. For instance, one male patient described experiencing a “tingling sensation in the head” as a warning sign. During public episodes, bystanders often mistake these events for epileptic seizures. However, patients who did not experience cataplexy do not exhibit muscle jerks, and the episode typically resolves on its own. On the other hand, medical assistance may be required in cases where the individual sustains an injury due to a sudden fall.

“In the year 2016/17, I started to deal with [narcolepsy], because I also started to have cataplexy, and whenever we were watching TV with the children, I got it, and it… it’s like paralysis, I can’t move my hands simply… my mother-in-law thought, that I was having a stroke, and she wanted to call me an ambulance, asking what’s going on, because I couldn’t speak, move… An ambulance was called to the store a few times, because I was also lying down. But it’s a paradox that when you get cataplexy, it’s like you get paralysis, but you hear everything they say, because we already tried the example at home, that when I woke up, I told them about what they were talking about.” *(F49NT1a)*.

#### Emotions triggering cataplexy

The individuals we interviewed identified positive emotions as triggers for cataplexy, with laughter being frequently mentioned. For example, one patient experienced his first cataplectic attack at a shopping mall after hearing a good joke. He later discovered he could induce cataplexy on his own by simply thinking about something funny. Some patients also mentioned negative emotions as triggers. Some reported that crying could trigger attacks, but fear could not. On the other hand, one patient found that fear and being scared were also triggers for him. Moreover, one patient described experiencing cataplexy during moments of yelling or arguing. Some patients experience cataplexy exclusively while feeling a negative emotion, which they find fortunate, because of the possibility of experiencing happiness and positive situations without the fear of a possible cataplexy attack.

“You won’t believe what can trigger emotions. People often talk about emotions as positive or negative, but I think, and I’m no psychologist, that the psychological term is more about stimuli or arousal. Some theories describe it as either positive or negative arousal. I realized back then that both positive and negative emotions can have the same effect. For example, I was walking, either with my girlfriend or alone, and a stunningly attractive woman walked by. You make eye contact, and then it happens again—and I went weak in the knees, no exaggeration. The same thing could happen with a guy walking by, giving you a hostile, tough guy look. You meet his gaze confidently, but again, I end up almost collapsing. It’s fascinating how such simple moments can have such a strong physical impact.” *(M40NT1)*.

#### Situations triggering cataplexy

Similar to their descriptions of emotions or arousal, the patients we interviewed also discussed specific situations that triggered cataplexy. For some, these episodes were associated with particular locations, but many reported experiencing them in contexts such as work or celebrations, i.e., in social situations. These situations often involved the previously mentioned positive or negative emotional stimuli. For instance, one patient experienced a cataplectic episode during a visit to her neurologist’s office, triggered as she entered the consultation room. She attributes this to nervousness about the uncertainty of the examination. Certain scenarios, however, pose significant safety risks. Some respondents experienced cataplexies on stairs, nearly resulting in falls. A young female patient recounted an episode occurring in the sea, a situation that could have led to drowning. Sexual intercourse was also a trigger for one female type one patient.

“For me that was already the second time, and a person doesn’t even notice it – I was standing in church, stiff and upright. A person falls asleep while standing, stiff and still, and then suddenly there’s a thud, and they’re on the ground. So, for me, it’s quite extreme: I’m somewhere, I stiffen up, and then I fall. It’s like that… but it hasn’t been happening as much lately. I’ve somehow stabilized it. Before, it happened more often when I went out into social settings. When I was overwhelmed or in a group of people, as the doctor explained, I could emotionally ‘discharge’ it. But now I’m in a kind of energy-saving mode, both emotionally and physically, so it has decreased. I’ve never been a party boy, never liked it. I didn’t go to discos or anything like that. But when I attended celebrations or events, where there were emotional excitement, I had more such experiences. But they’ve been sporadic throughout my life – completely random. For example, it would happen on the bus, especially when I was with friends, talking or something like that. Or in church – that’s interesting because I didn’t have anyone there; you’re just listening to the mass, and yet you end up having such an experience. Now that I think about it, I didn’t experience it much in school, probably because I was often sitting at a desk.” *(M39NT1)*.

#### Factors influencing cataplexy

The manifestation of cataplexy is influenced by both the experienced emotion and the context in which the individual finds himself. Participants in our study provided insights into factors beyond emotional experiences that can affect the frequency and intensity of cataplectic episodes. One recurring theme was the inability to control overwhelming emotions, where patients feel compelled to surrender to the emotional experience, subsequently triggering cataplexy. Some participants suggested that psychological resilience plays a significant role in managing cataplexy. One patient described learning to suppress emotional responses entirely, stating that he now reacts in an emotionally detached manner. Other participants reported that cataplexy worsens when they attempt to resist fatigue. Suppressing tiredness and drowsiness during a church service led one patient to her knees giving way and falling.

“If I didn’t take the medication, I can’t imagine how I would function because you know… the medication I take for cataplexy and narcolepsy suppresses both conditions. And if I didn’t, for example, take the medication for cataplexy, I’d feel it more… I’d constantly feel that sensation. I’d be more sluggish, dizzy—not exactly… I wouldn’t be as alert, but rather a bit dazed. That’s what it does, this strange feeling, like when you are kind of drowsy or about to fall asleep. It’s this dazed, unpleasant feeling, combined with a headache. And when you experience emotions, it suddenly flares up—like when you laugh, for example. That’s when cataplexy happens, and on top of that, your legs give way; you get so weak that you must be careful not to fall. All this together, and that’s what the medication suppresses.” *(F22NT1)*.

#### Changes of cataplexy in time

Despite cataplexy being one of the hallmark symptoms of NT1, its frequency and presentation can vary significantly among patients. In some individuals, cataplexy occurs only rarely, while others experience it daily. One patient reported that her episodes of cataplexy are infrequent and tend to manifest during particularly challenging life situations or specific emotional states, such as at funerals. Conversely, another patient experienced extreme manifestations at the beginning of his sickness but now can manage it effectively.

“But the first years, the initial period after it was all diagnosed, were probably the hardest. The cataplexy was severe—intense—and I dealt with it daily, sometimes 50–60 episodes. I had these attacks where, if I laughed even a little or felt any emotion, I would just collapse. It happened countless times. For example, if I was walking down the street and a funny thought came to mind, even if no one else knew or I laughed internally, I’d already be lying somewhere in the middle of the road. So, the early stages of this illness were very difficult.” *(M44NT1)*.

In many patients, the symptoms of narcolepsy evolve throughout the disease. Numerous factors influence these changes, including how long the patient has lived with the condition, how accustomed they have become to the symptoms, and their ability to manage them. Treatment and medication usage also play a significant role. For some patients, symptoms improve following diagnosis and initiation of treatment, to the extent that they cannot imagine functioning without medication. However, it is common for individuals with narcolepsy to seek professional help only when their symptoms intensify or worsen. In some cases, even after treatment is introduced, there is no noticeable improvement, and symptoms may even deteriorate over time.

“[The triggers] now it’s only negative [emotions] ones. Before, it used to be just positive—like when I laughed heartily or felt joy about something. But now I’ve noticed that it also happens when something extremely upsets me.” *(F28NT1)*.

#### Emotional experience with cataplexy

Patients commonly describe cataplexy as a highly distressing experience, with its impact often influenced by the circumstances in which an episode occurs. Many report that cataplexy is particularly uncomfortable when it happens in public or around strangers. One patient mentioned the discomfort of experiencing cataplexy primarily in her legs, which forces her to quickly call someone for assistance to prevent falling. Another one, a physician, shared how challenging it is during her hospital rounds when patients make humorous remarks, causing her knees to give way. Others described the unpleasant sensation of trying to suppress an episode of cataplexy, which often results in a severe headache.

“So, it’s mostly laughter or joy—you know, you go somewhere, start laughing, or someone tells you a joke, and you laugh, or something happens. We humans can be a bit malicious, as you might say. For example, if someone falls; the poor person is all bruised up, but at that moment, not just you but the whole group or those around you find it funny. Only later does it hit them, ‘Oh my gosh, I’m sorry we laughed; it must have hurt so much, or you must’ve injured yourself.’ These emotions, you see. And when it happens in an unfamiliar environment, it’s very uncomfortable. Even within the family, they never really got used to it. It’s always like, ‘Why are you making that face now? Why are you pulling such expressions? What’s this or that all about?’” *(F52NT1)*.

### Nocturnal sleep

Nighttime sleep in patients with narcolepsy can vary widely. For some, it is deep and restorative, while for others, it is disrupted and fragmented, with frequent awakenings. For instance, an NT1 patient reported that she falls asleep quickly and sleeps well initially but often wakes up during the night. In contrast, both NT2 patients interviewed stated they fall asleep immediately, regardless of noise or light in their surroundings, and enjoy uninterrupted sleep. Although excessive daytime sleepiness is a hallmark symptom of narcolepsy, nighttime sleep is not always ideal for these patients. Many report difficulties with nocturnal sleep, particularly concerning frequent awakenings. This sleep fragmentation can be influenced by various common stimuli. For example, an elderly female patient stated that she experiences significant difficulty falling asleep at night, to the extent that she needs to take sleeping medication. Meanwhile, another mentioned that she struggles to sleep during a full moon because the moonlight wakes her up.

“Well, sometimes I sleep, sometimes I don’t. My nighttime sleep starts off fine—I fall asleep quickly, which is good—but then I wake up very often. Aside from the usual trips to the bathroom, I just wake up frequently. Occasionally, I’ll have a good night, but that usually depends on several factors aligning: having a good workout and eating the right foods—not pizza or anything like that. When everything aligns, I might only wake up once or twice in the night. But that’s rare—maybe two or three nights out of ten, if even that. It might just be a coincidence when it happens. Most of the time, my sleep is very disrupted. I’m constantly tossing and turning, changing positions, or sometimes just lying there staring, knowing I can’t keep lying awake like that. I must do something to get back to sleep so I can function the next day. Overall, my sleep could be better.” *(M29NT1)*.

Patients also mentioned certain behaviors occurring during nighttime sleep. For some patients also suffering from obstructive sleep apnea (OSA), it is usual that they experience snoring. One patient also mentioned that she experiences whispering during nocturnal sleep and rolling on the bed uncomfortably.

“[…] when my boyfriend slept next to me recently, he said that I grind my teeth. Sometimes I catch myself doing it, yeah, grinding my teeth. And I wouldn’t say that I snore much. But he says I snore a lot and that I have whole conversations with myself.” (*F26NT1b)*.

### Dreams and nightmares

#### Vivid dreams

The dreams experienced by individuals with narcolepsy can be extraordinarily vivid and, as they describe, exceptionally lifelike. These dreams often take place in realistic scenarios, though they may sometimes occur in imaginary worlds or involve scenes from movies they fell asleep watching. When patients spoke about “vivid dreams,” they mentioned struggling to distinguish between what they had dreamed and what had happened. Some respondents described these dreams as action-packed and colorful. These dreams are often repetitive, with some patients reporting that the dreams are interconnected, continuing their narrative after awakening and falling back to sleep. A male patient experiences vivid dreams so frequently that he has learned to control them, consciously interacting with characters or observing the storyline. Such dreams are referred to as lucid dreams or wakeful dreams.

“Well, I experience a lot of lucid dreams, and it kind of developed gradually. I mainly have them during naps, for instance. This is one of the more interesting aspects of the condition, though I would, of course, trade it in a heartbeat to not have the condition at all. What is particularly fascinating is that I’m much more aware of the gradual transition—I see the entire process of falling asleep because I remain fully conscious throughout the nap. These naps often transition into lucid dreams for me. So, it’s quite interesting that I can have two or three lucid dreams a day, where I’m fully aware of the dream and can control it. Another thing I’ve noticed is how incredibly convincing these dreams are. I have a scientific outlook on the world, and yet, because you’re in this wakeful state during the dream, everything feels very real—like reality itself. It’s as if you perceive things in a heightened way. I can imagine that for some people, this could even alter their perspective on objective reality, leading them to believe in certain things they wouldn’t otherwise.” *(M37NT1)*.

#### Content of the dreams

Narcoleptic dreams can vary widely in content. Many patients describe a strong connection between dreams and reality. Some patients also report that the content of their dreams can be frightening or even nightmarish. These dreams are often extremely vivid and detailed, resembling scenes from a movie. Notably, such dreams may occur not only during nighttime sleep but also during episodes of EDS. One patient recounted a negative experience with nightmares only during his teenage years. He described dreams where his entire family was dead or where he was being chased by a skeleton. Another patient shared she was dreaming about spiders and snakes, animals she greatly dislikes.

“The dreams I have are often connected to spiritual and supernatural themes, which is surprising because I never really focus on such things. Sure, I might say a prayer before bed and attend church on Sundays, but it’s strange how often I dream about supernatural things. It’s unusual. […] dreams about heaven, clouds, angels, and things like that. It feels odd to me because I don’t typically think about such things. Even when I draw or look for inspiration online, I don’t paint things like that. Maybe when I’m in church, during the service, I reflect and think while listening to the sermon, but I’m never really focused on it to the extent that I’d expect to have supernatural dreams. Yet sometimes I do. And it’s not like I read the Bible or anything; I only hear the sermons in church.” *(F33NT1)*.

#### Factors influencing dreams

Dreams can be influenced by various factors, such as daily experiences, emotions, heightened stress, or anxiety. In the case of narcolepsy patients, their experiences suggest that increased daytime fatigue or EDS, medication-related fluctuations, or even watching horror movies can affect the content of their dreams, often resulting in these themes appearing during nighttime sleep.

“I avoid watching horror movies entirely because I believe they would influence the themes of my dreams. I don’t watch them at all, yet these types of dreams occur regularly. Honestly, I could probably create a horror movie or write a book based on them.” *(F337NT1)*.

### Hallucinations

#### Modality of hallucinations

Many patients experience hallucinations during the night, which often occur just before falling asleep or immediately upon waking. This timing can make it challenging to discern whether these are hallucinations or vivid dreams. One patient describes them as scenarios where she finds herself in a dream but is unsure if she has woken up, leading her to identify them as hallucinations. Hallucinations can take on various forms, including tactile, visual, and auditory. Experiences of auditory hallucinations, such as hearing voices and footsteps, are often accompanied by an inability to move. Another described a moment of desperation when she attempted to pray but felt as though something was holding her hands apart, preventing her from bringing them together. Some people experience similar patterns in what they perceive at that moment. Many patients talked about black figures present in the room with them, sometimes even in their immediate proximity. Some stated the hallucinated scenes as being like a movie they fell asleep watching.

“At first, it was just like touch, like someone was touching me or pulling my hair, and then, or rather, touching me in a spot that would then burn intensely. But later, I started hearing footsteps in the apartment all the time, or ringing, and I couldn’t wake up at that moment because it was also connected to cataplexy. When I finally managed to wake up and ran to open the door, no one was there, but I would even open the door at night. It didn’t even occur to me that no one could ring at our place. Then, at night, I’d feel someone moving around the bed. For instance, I could hear someone walking around the bed, and we had parquet flooring with a carpet on top, so I knew exactly where it creaked, and where the person probably was. Later, we replaced the flooring with laminate, and I stopped hearing the creaking of the carpet and started hearing soft steps instead. […] I haven’t heard voices, except for one really unpleasant time. Once, it seemed like someone was standing in the doorway watching me, and I got so scared that I went to hide in my daughter’s bed because my husband wasn’t home. So, I crawled into my daughter’s bed. That was the only time I heard voices—they said, “Kill her, because she’s the cause of all the bad things happening to you.” That really terrified me, and we ran away to sleep at the neighbors.” *(F50NT1)*.

#### Factors influencing hallucinations

Whether hallucinations manifest in a patient can depend on several factors. During interviews, patients mentioned a few of these. Some patients said the hallucinations occur when they forget to take their medication. They also tend to intensify when they try to forcibly wake themself from the hallucination. That’s why she tells herself to stay still and pretend to be asleep. She convinces herself that it’s just a dream and that she needs to let it pass on its own. Hallucinations may also occur during EDS or daytime naps.

“Even when I go [to sleep] very tired, exhausted and late, I also have, as they say, such morbid dreams that I fall asleep, and it seems to me that something… I can’t scream, I can’t talk, and I prefer I would scream for someone to help me, I feel like something is dragging me around the room, beating me, also when I’m tired. I am terribly afraid then. I’m scared, I can’t breathe.” *(F49NT1b)*.

### Sleep paralysis

Hallucinations can be present in narcolepsy as a separate manifestation of the disease but are often described by patients in connection with sleep paralysis. This is a condition that occurs when suddenly falling asleep or waking up, when the body is unable to perform movements. At that time, the person is conscious but can only move their eyes and breathe. In this process, the already mentioned black figure or other hallucinations or visions can be perceived. Some patients described an inability to scream during the episode. Some patients mentioned they felt someone lying in bed next to them.

“I now know that when I’m falling asleep, it comes in waves, and then I become alert, waiting for it to happen. At that point, fear sets in, my heart starts racing, and I feel like my brain gets more blood flow, which seems to trigger those images and sounds. When the waves start, I tell myself, ‘Something’s coming, don’t move, pretend you’re asleep.’ I try not to acknowledge it, convincing myself it’s just a dream, and that it will pass. The worst thing is trying to wake up from it— the more I tried to wake myself, the harder it became. Breathing also got more difficult during those moments. For example, when I was married, my husband became used to it. Whenever I experienced sleep paralysis, I could at least manage to breathe more audibly. If he hadn’t fallen asleep yet, he’d recognize it and know to wake me up. He was able to wake me, but I couldn’t do it myself. The harder I tried, the worse it got. It’s better to just let it pass, and eventually, it fades away.” *(M50NT1)*.

### Emotional experiences with sleep impairments

Most patients agreed that experiencing the symptoms we describe (nocturnal sleep, dreams and nightmares, hallucinations, and sleep paralysis) is an unpleasant and negative experience. Some patients claimed those experiences were unpleasant mainly because of the nightmares, from which they wake up screaming, and that they still remember them all. Nightmares were also described as “disgusting”. One patient described these experiences as “mentally demanding” and another as “annoying” because he wakes up in the night and cannot get a good night’s sleep. Some described them as “crazy”, but some patients even thought they were “going crazy” due to parasomnias and should seek psychiatric help.

“Nightmares, well, bad dreams. I sleep with the light on; I can’t sleep in the dark, so you get stiff, you get stiff during sleep, you can’t move, and you see something. Or I don’t know, you can’t even shout or do anything. You just completely freeze.” *(F52NT1)*.

“For example – even my girlfriend laughs about it – that it’s just me sleeping with the door locked and even if I just hear something, that I hear some steps maybe and so on, I don’t care in quotes. Because I simply have the door locked, I know that no one will simply get in, so I somehow logically substantiated it, that I am simply safe, no one will come there, nothing can happen there, so it’s fine.” *(M29NT1)*.

## Discussion

This study aimed to understand and further describe the perception of narcolepsy patients on symptomatology using a qualitative approach with semi-structured narrative interviews. This study is part of a bigger project focused on the overall perception of patients’ experiences with this disease, and this paper is one part of these outcomes. In this paper, we described the main symptoms of narcolepsy through the perceptions and emotional experiences of the patients – excessive daytime sleepiness, cataplexy, nocturnal sleep, dreams and nightmares, hallucinations and sleep paralysis – using narrative thematic analysis [23, 24]. We found that patients’ perspectives describe the symptoms more deeply compared to diagnostic manuals and the literature. These experiences also varied in multiple aspects compared among patients, e.g., the frequency and intensity of manifestation, influencing factors or occurrence, and its changes throughout the lifespan with the disease.

EDS was often described by patients as the experience of “microsleeps” or a sensation of being “shut off.” While the manifestation was typically uncontrollable, some patients reported premonitory sensations, such as discomfort in the eyes or incoherent speech, allowing them to act before EDS onset. However, patients sometimes conflated EDS with fatigue or nocturnal sleep, and their descriptions frequently employed non-standard terminology. Patients do not necessarily need to understand whether the practitioner is asking about EDS or fatigue and combines these terms together. As Bassetti et al. [12] found, patients suggest the medical doctors to use more open-ended not leading questions with the focus on the EDS, cataplexy yet al.so other aspects outside the core of the narcolepsy symptomatology to fully understand the sleep-wake disorders. These inconsistencies in terminology and expressions may also contribute to prolonged diagnostic delays, misdiagnosis, or lack of recognition by general practitioners. Therefore, the term “energy fluctuation” might be used in this matter and should be considered in the field of experts while talking with the patients. The average diagnostic delay for narcolepsy is approximately seven years. Factors such as the absence of cataplexy, comorbid conditions, and limited awareness of narcolepsy’s symptomatology among both patients and clinicians exacerbate these delays [25]. Notably, the lack of knowledge regarding narcolepsy is not confined to the general population but extends to healthcare professionals, including clinicians, physicians, and even some sleep specialists [26].

Cataplexy is a potentially health- and well-being-threatening symptom, primarily associated with the diagnosis of NT1. In this study, patients typically did not experience cataplexy simultaneously with EDS, delaying their decision to seek medical help until cataplexy onset. While some patients experienced partial manifestations, such as arm or mouth drooping, others reported generalized muscle weakness. Barateau et al. [27] observed that certain attacks may begin as partial and progress to generalized muscle weakness, while others remain partial, though the underlying mechanism of this phenomenon remains unknown. The patients in this study described cataplexy in relation to perceived influencing factors. Commonly, it was triggered by positive emotions or situations, such as laughter or joyful moments, but some also reported negative triggers, including arguments or screaming. Additional factors, such as psychological and physical resilience, were noted, and one patient identified sex as a trigger for cataplexy. This phenomenon, termed “orgasmolepsy,” can significantly impact a patient’s sexual, social, and emotional life due to its potential for embarrassment [28]. Poryazova et al. [29] documented similar cases, noting that cataplexy during sexual activity was rare and often influenced by emotional commitment and trust in relationships. Moreover, they highlighted that this symptom could occur in other sleep-related disorders, not exclusively narcolepsy.

Other symptoms experienced by narcolepsy patients are often characterized as sleep impairments. Disrupted nocturnal sleep was prevalent among patients with NT1 but less so in those with NT2. These disturbances were frequently associated with vivid dreams and nightmares, which patients often described as disruptive. One patient reported the ability to engage in lucid dreaming. Factors such as medication, increased fatigue, or exposure to horror films were noted as potential influences on these dreams. Pisko et al. [30] reported in their study sample a prevalence of 36% mundane dreams, 26% vivid but not unpleasant dreams, and 33% nightmares, with 5% experiencing a reduction in nightmares. Nightmares were described as “dreadful, horror-like, and terrifying,” suggesting they may significantly affect the overall experience of narcolepsy, sleep quality, and QoL. Dreams and nightmares in the study were often interchanged with symptoms of hypnagogic hallucinations and sleep paralysis. Hallucinations were described as sensory experiences, including visual (e.g., seeing dark figures), auditory (e.g., hearing sounds), and tactile (e.g., feeling restrained). During these episodes, patients often did not distinguish between hallucinations and sleep paralysis, as they were frequently reported to occur simultaneously.

A recent Australian study by Shockman et al. [16] similarly explored the perception of narcolepsy symptoms, examining multiple aspects, including EDS, cataplexy, the disease’s impact on daily life, and long-term care. In contrast, our study concentrated specifically on narcolepsy symptomatology, delving deeper into the nuances of each symptom through a focused analysis rather than relying on broader patient narratives. This approach offers a more detailed and specific insight into the phenomenon of narcolepsy symptomatology. Interestingly, despite geographical and cultural differences, the findings from the Australian study align closely with the experiences reported by Slovak narcolepsy patients, particularly in the perception and description of symptoms. Shockman et al. [16] found that patients often grouped four symptoms—fatigue, lack of energy, sleepiness, and being sleepy—under the umbrella of EDS. Similarly, our findings revealed that Slovak patients frequently used comparable terms to describe EDS and often failed to distinguish it from nocturnal sleep.

Moreover, our study adds a unique dimension by providing insights into the emotional experiences associated with EDS, cataplexy, and other sleep impairments, offering a more comprehensive understanding of these symptoms. EDS and cataplexy were predominantly perceived negatively due to their social consequences, societal prejudices, and associated feelings of confusion and shame. However, some patients reported EDS as pleasant in situations where resisting the urge to sleep was unnecessary. Sleep disruptions and parasomnias were described as profoundly negative and disruptive, with some patients initially believing they had a psychiatric condition, such as schizophrenia. The emotional impact of these symptoms was reflected in patient behavior; for instance, one patient reported needing the light on to sleep, while another locked their bedroom door to feel secure and differentiate parasomnias from reality. Schiappa et al. [31] suggest that the emotional aspects of sleep impairments may be linked to the involvement of the limbic system and amygdala in cataplexy and nocturnal sleep disturbances in narcolepsy. They also highlight difficulties in emotional processing and coping mechanisms among patients. However, the relationship between narcolepsy and emotional processing remains poorly understood. On the other hands, the patients recommend the necessity of specialized nurses and psychologist to be working at the sleep clinics to fill the gap in the needs of the patients, addressing their issues and help management, together with practical patients’ concerns [12]. Therefore, there is an emerging necessity to specify the diagnostic manuals (e.g., ICSD-3-TR) more deeply with the incorporation of patients’ experiences. First, this might include anonymized recent case studies for comparison with patients to humanize the disease and point out its impact on patients’ lives. Second, the description of the symptoms impact on emotional experiences and daily life could be added, together with the social, cultural and economic impacts of the disease. Third, the Patient-Reported Outcome Measures (PROMs) could be helpful in integrating the patients’ perspective in the measurements of the symptoms.

### Strengths and limitations

A notable strength of this study lies in its rigorous methodological framework [19] and collaborative analytical process. Multiple authors contributed to the coding, analysis, and interpretation of results. Objectivity was enhanced through individual coding, which was subsequently refined through consensual agreements among the authors during each phase of the analysis. This methodological rigor supports the credibility of the study’s findings. Therefore, findings from this study could potentially be considered in the treatment planning and therapy establishing.

To our knowledge, no recent qualitative studies have specifically examined patients’ perceptions of narcolepsy symptoms. Existing research employing qualitative approaches has focused primarily on specific aspects, such as cataplexy [32] or the management of narcolepsy in a single patient [17], or targeted specific subgroups, including adolescents [33] and female patients [34].

Although this study represents the first qualitative investigation of Slovak narcolepsy patients, it also has certain limitations. The sample reflects the sociodemographic characteristics of the Slovak narcolepsy population, but the disproportion between NT1 and NT2 patients precluded a comprehensive analysis of differences in narratives and perspectives between these groups. Nevertheless, the sample size represents nearly half of the Slovak narcolepsy population, calculated based on the most recent database published in 2020 [7]. It should also be noted that not all patients included in the study were undergoing pharmacological treatment. Only 11 patients (44% of the sample) were medicated at the time of the interviews. One patient declined medication despite suboptimal symptom management. Several other patients were not receiving pharmacotherapy, instead managing their symptoms through lifestyle modifications. Some patients were withdrawn from medication because Pitolisant is not fully reimbursed and the surcharge from the patient is too high. Importantly, no significant impact of treatment status on word fluency, motivation, or recall was observed during the interviews. Furthermore, potential inaccuracies were minimized through the validation process, as patients were given the opportunity to review and correct their verbatim interview transcripts. Additionally, only accurately diagnosed patients were included, ensuring a high level of diagnostic validity.

## Conclusion

The importance of qualitative research in narcolepsy has been highlighted by Kapella et al. [35], who emphasized that qualitative methodologies could offer a deeper understanding of the condition by capturing the lived experiences of patients. The need for qualitative studies is increasingly evident when addressing the unique challenges faced by rare patient populations. As is shown in our study, the burden of narcolepsy symptoms might be better perceived by patients in their own words rather than assessed using questionnaires. Exploring narcolepsy symptomatology from the patient’s perspective could facilitate advancements in treatment approaches that extend beyond symptom suppression to address the psychological and social dimensions of living with this condition.

## Data Availability

The data from this research cannot be shared due to ethical and privacy reasons of each interviewed individual participating in the study.

## References

[1] American Academy of Sleep Medicine (203). *International Classification of sleep disorders (3rd edition, text revision).* Darien, IL: American Academy of Sleep Medicine

[2] Sateia MJ (2014) International classification of sleep Disorders – Third edition: highlights and modifications. Chest 146(5):1387–1394. 10.1378/chest.14-0970 25367475 10.1378/chest.14-0970

[3] Bassetti, C.L.A., Adamantidis, A., Burdakov, D.,… Dauvilliers, Y. (2019). Narcolepsy – clinical spectrum, aetiopathophysiology, diagnosis and treatment. Nature Reviews Neurology, 15, 519–539. 10.1038/s41582-019-0226-9 10.1038/s41582-019-0226-9 31324898

[4] Barateau L, Morse AM, Gill SK, Pizza F, Ruoff C (2024) Connecting clinicians and patients: the Language of narcolepsy. Sleep Med 124:510–521. 10.1016/j.sleep.2024.10.014 39437461 10.1016/j.sleep.2024.10.014

[5] Baker EC, Flygare J, Paruthi S, Sharkey KM (2020) Living with narcolepsy: current management strategies, future prospects, and overlooked Real-Life concerns. Nat Sci Sleep 12:453–466. 10.2147/NSS.S162762 32765142 10.2147/NSS.S162762 PMC7371435

[6] Partinen M, Kornum BR, Plazzi G, Jennum P, Julkunen I, Vaarala O (2014) Narcolepsy as an autoimmune disease: the role of H1N1 infection and vaccination. Lancet Neurol 13(6):600–613. 10.1016/s1474-4422(14)70075-4 24849861 10.1016/S1474-4422(14)70075-4

[7] Feketeova E, Tormasiova M, Klobučníková K, Durdik P, Jarcuskova D, Benca M, Vitkova M (2020) Narcolepsy in Slovakia – epidemiology, clinical and polysonographic features, comorbid diseases: A case-control study. Sleep Med 67:15–22. 10.1016/j.sleep.2019.10.012 31884306 10.1016/j.sleep.2019.10.012

[8] Kallweit U, Schmidt M, Bassetti CL (2017) Patient-Reported measures of narcolepsy: the need for better assessment. J Clin Sleep Med 13(5):737–744. 10.5664/jcsm.6596 28162143 10.5664/jcsm.6596 PMC5406952

[9] Johns MW (1991) A new method for measuring daytime sleepiness: Epworth sleepiness scale. Sleep 14(6):540–545. 10.1093/sleep/14.6.540 1798888 10.1093/sleep/14.6.540

[10] Sturzenegger C, Baumann CR, Lammers G, Kallweit U, van der Zande W, Bassetti CL (2018) Swiss narcolepsy scale: a simple screening tool for hypocretin-deficient narcolepsy with cataplexy. Clin Translational Neurosci 2(2):1–5. 10.1177/2514183x18794175

[11] Lammers, G.J., Bassetti, C.L., Dolenc-Groselj, L.,… Dauvilliers, Y. (2020). Diagnosis of central disorders of hypersomnolence: A reappraisal by European experts. Sleep Medicine Reviews, 52, 101306. 10.1016/j.smrv.2020.101306 10.1016/j.smrv.2020.101306 32311642

[12] Bassetti, C.L.A., Kallweit, U., Vignatelli, L.,… Lammers, G.J. (2021). European guidelines and expert statements on the management of narcolepsy in adults and children. European Journal of Neurology, 28(9), 2815–2830. 10.1111/ene.14888 10.1111/ene.14888 34173695

[13] Wasling HB, Bronstein A, Wasling P (2020) Quality of life and procrastination in post-H1N1 narcolepsy, sporadic narcolepsy and idiopathic hypersomnia, a Swedish cross-sectional study. Sleep Med 76:104–112. 10.1016/j.sleep.2020.10.014 33152582 10.1016/j.sleep.2020.10.014

[14] Chin W-C, Wang C-H, Huang Y-S, hsu J-F, Chu K-C, Tang I, Paiva T (2022) Quality of life changes and their predictors in young adult narcolepsy patients after treatmnent: A real-world cohort study. Front Psychiatry 13:956037. 10.3389/fpsyt.2022.956037 36016973 10.3389/fpsyt.2022.956037 PMC9395703

[15] Ong JC, Fox RS, Brower RF, Mazurek S, Moore C (2020) How does narcolepsy impact Health-Related quality of life?? A Mixed-Methods study. Behav Sleep Med 19(2):145–158. 10.1080/15402002.2020.1715411 31937147 10.1080/15402002.2020.1715411

[16] Schockman A, Cheung J, Milton A, Naehrig D, Thornton N, Bin YS, Kairaitis K, Glozier N (2024) Making sense of narcolepsy: A qualitative exploration of how persons with narcolepsy percieve symptoms and their illness experience. Sleep Med 116:62–70. 10.1016/j.sleep.2024.02.026 38430792 10.1016/j.sleep.2024.02.026

[17] Franceschini C, Fante C, Filardi M, Folli CM, Brazzi F, Pizza F, D’Anselmo A, Ingravallo F, Antelmi E, Plazzi G (2020a) Can a Peet support the process of Self-Management in narcolepsy?? A qualitative narrative analysis of a narcoleptic patient. Front Psychol 11:1353. 10.3389/fpsyg.2020.01353 32733314 10.3389/fpsyg.2020.01353 PMC7358570

[18] Tadrous R, O’Rourke D, Murphy N, Quinn G, Qiunn C, Slattery L, Broderick J (2024) Exploring exercise, physical wellbeing and the role of physiotherapy: perspectives from people with narcolepsy. J Sleep Res 33(2):e14007. 10.1111/jsr.14007 37621198 10.1111/jsr.14007

[19] Ziebland S, McPherson A (2006) Making sense of qualitative data analysis: an introduction with illustrations from DIPEx (personal experiences of health and illness). Med Educ 40:405–414. 10.1111/j.1365-2929.2006.02467.x 16635119 10.1111/j.1365-2929.2006.02467.x

[20] Ziebland S, Herxheimer A (2008) How patients’ experiences contribute to decision making: illustrations from DIPEx (personal experiences of health and illness). J Ursing Manage 16:433–439. 10.1111/j.1365-2834.2008.00863.x 10.1111/j.1365-2834.2008.00863.x 18405260

[21] Ziebland S, Wyke S (2012) Health and illness in a connected world: how might sharing experience on the internet affect people’s health?? Milbank Q 90(2):2019–2249. 10.1111/j.1468-0009.2012.00662.x 10.1111/j.1468-0009.2012.00662.x PMC3460203 22709387

[22] Hurley E, Dietrich T, Rundle-Thiele S (2021) Integrating theory in Co-design: an abductive approach. Australasian Mark J 29(1):66–77. 10.1177/1839334921998541

[23] Ross JA, Green C (2011) Inside the experience of anorexia nervosa: A narrative thematic analysis. Counselling Psychother Res 11(2):112–119. 10.1080/14733145.2010.486864

[24] Clarke V, Braun V (2017) Thematic analysis. J Posit Psychol 12(3):297–298. 10.1080/17439760.2016.1262613

[25] Bhattarai J, Sumerall SW (2018) Diagnostic delay of narcolepsy: contributing factors and implications for clinicians. Sleep Vigilance 2:103–109. 10.1007/s41782-018-0046-9

[26] Rosenberg R, Kim AY (2014) The AWAKEN survey: knowledge of narcolepsy among physicians and the general population. Postgrad Med 126(1):78–86. 10.3810/pgm.2014.01.2727 24393754 10.3810/pgm.2014.01.2727

[27] Barateau L, Pizza F, Lopez R, Antelmi E, Plazzi G, Dauvilliers Y (2018) Persistence of deep-tendon reflexes during partial cataplexy. Sleep Med 45:80–82. 10.1016/j.sleep.2017.12.011 29680434 10.1016/j.sleep.2017.12.011

[28] Pellitteri G, Dolso P, Valente M, Gigli GL (2020) Orgasmolepsy in narcolepsy type 1 responsive to pitolisant: A case report. Nat Sci Sleep 12:1237–1240. 10.2147/NSS.S286358 33408544 10.2147/NSS.S286358 PMC7780985

[29] Poryazova R, Khatami R, Werth E, Bassetti CL (2009) Weak with sex: sexual intercourse as a trigger for cataplexy. J Sex Med 6(8):2271–2277. 10.1111/j.1743-6109.2009.01328.x 19493288 10.1111/j.1743-6109.2009.01328.x

[30] Pisko J, Pastorek L, Buskova J, Sonka K, Nevsimalova S (2014) Nightmares in narcolepsy: underinvestigated symptom? *Sleep Medicine, 15*(8), 967–972. 10.1016/j.sleep.2014.03.006Franceschini, Ch., Fante, Ch., Folli, C.M., Filosa, M., Pizza, F., Antelmi, E., Ingravallo, F, & Plazzi, G. (2020b). Giving a voice to cataplectic experience: recollections from patients with narcolepsy type 1. *Journal of Clinical Sleep Medicine, 16*(4), 597–603. https://doi.org/10.5664/jcsm.8286 10.5664/jcsm.8286 PMC7161466 32022668

[31] Schiappa C, Scarpelli S, D’Atri A, Gorgoni M, Gennero LD (2018) Narcolepsy and emotional experience: a review of the literature. Behav Sleep Med 22(2):179–189. 10.1080/15402002.2023.2217971 10.1186/s12993-018-0151-x PMC6305999 30587203

[32] Franceschini C, Fante C, Folli CM, Filosa M, Pizza F, Antelmi E, Ingravallo F, Plazzi G (2020b) Giving a voice to cataplectic experience: recollections from patients with narcolepsy type 1. J Clin Sleep Med 16(4):597–603. 10.5664/jcsm.8286 32022668 10.5664/jcsm.8286 PMC7161466

[33] Chen A, Parmar A, Naik T, Hutchinson C, Frankel L, Zweerink A, Murray BJ, Toulany A, Narang I (2021) Living with narcolepsy during adolescence: A qualitative study. Can J Respiratory Crit Care Sleep Med 6(3):161–168. 10.1080/24745332.2021.1899088

[34] Kilmartin B, Day W (2024) It’s like tumbleweeds everywhere’: an interpretative phenomenological analysis of the lived experiences of being diagnosed with and living with narcolepsy. J Health Psychol 29(12):1336–1349. 10.1177/13591053231221373 38284414 10.1177/13591053231221373 PMC11459859

[35] Kapella MC, Berger BE, Vern BA, Vispute S, Prasad B, Carley DW (2015) Health-Related stigma as a determinant of functioning in young adults with narcolepsy. PLoS ONE 10(4):e0122478. 10.1371/journal.pone.0122478 25898361 10.1371/journal.pone.0122478 PMC4405359

